# Carrier Depletion near the Grain Boundary of a SiC Bicrystal

**DOI:** 10.1038/s41598-019-54525-z

**Published:** 2019-11-29

**Authors:** Young-Wook Kim, Eita Tochigi, Junichi Tatami, Yong-Hyeon Kim, Seung Hoon Jang, Srivani Javvaji, Jeil Jung, Kwang Joo Kim, Yuichi Ikuhara

**Affiliations:** 1 Functional Ceramics Laboratory, Department of Materials Science and Engineering, The University of Seoul, Seoul, 02504 Republic of Korea; 2 Institute of Engineering Innovation, The University of Tokyo, Tokyo, 113-8656 Japan; 3 Graduate School of Environmental and Information Sciences, Yokohama National University, Yokohama, 240-9501 Japan; 4 Department of Physics, The University of Seoul, Seoul, 02504 Republic of Korea; 5 Department of Physics, Konkuk University, Seoul, 05029 Republic of Korea

**Keywords:** Engineering, Materials science

## Abstract

Silicon carbide (SiC) bicrystals were prepared by diffusion bonding, and their grain boundary was observed using scanning transmission electron microscopy. The n-type electrical conductivity of a SiC single crystal was confirmed by scanning nonlinear dielectric microscopy (SNDM). Dopant profiling of the sample by SNDM showed that the interface acted as an electrical insulator with a ~2-μm-thick carrier depletion layer. The carrier depletion layer contained a higher number of oxygen impurities than the bulk crystals due to the incorporation of oxygen from the native oxide film during diffusion bonding. Density functional theory calculations of the density of states as a function of the bandgap also supported these findings. The existence of a carrier depletion layer was also confirmed in a p-type polycrystalline SiC ceramic. These results suggest that the electrical conductivity of SiC ceramics was mostly affected by carrier depletion near the grain boundary rather than the grain boundary itself.

## Introduction

Silicon carbide (SiC) is an important structural ceramic because of its unique advantages, including excellent mechanical properties, oxidation and corrosion resistance at high temperatures, and high thermal conductivity^1–7^. In addition, SiC has received considerable attention as a high-temperature semiconductor for power electronics and optoelectronic applications because of its very wide bandgap and high-temperature stability^8–10^. Recently, a better understanding of the electrical properties of polycrystalline SiC ceramics has been achieved, including that of the N-doping mechanism in SiC ceramics by solution reprecipitation^11^, donor-acceptor compensation by co-doping with Al and N^12^, and N-doping promotion in SiC grains by the addition of nitrides (BN, Si_3_N_4_, TiN, and ZrN)^13–16^. However, systematic investigation of the effect of grain boundaries on the electrical conductivity of ceramics has been rarely reported. The electrical conductivity of polycrystalline SiC has been shown to be affected by the grain boundary phase composition^17^ and the segregation of atoms at grain boundaries^18^. All previous studies^17–20^ have investigated the boundary effects in polycrystalline materials or at the interfaces between different materials. Although the investigation of electrical conductivity across a single boundary of the same material is fundamentally and practically important to understand the effect of grain boundaries on the electrical conductivity of polycrystalline materials, no study has been conducted on this effect.

In this study, we prepared SiC bicrystals by diffusion bonding^21,22^ and characterized their grain boundary structures using scanning transmission electron microscopy (STEM). The carrier distribution near the grain boundary of a SiC bicrystal was investigated using SNDM. Density functional theory (DFT) calculations were conducted to examine all the possible cases of either oxygen or nitrogen doping in the SiC lattice. To expand the current results to polycrystalline SiC ceramics, a polycrystalline SiC ceramic was prepared by hot-pressing a SiC powder mixture containing 1 vol% Y_2_O_3_-Sc_2_O_3_ additives. The carrier distribution near the grain boundaries in the SiC polycrystal was also investigated using SNDM.

## Results and Discussion

**Figure 1:**
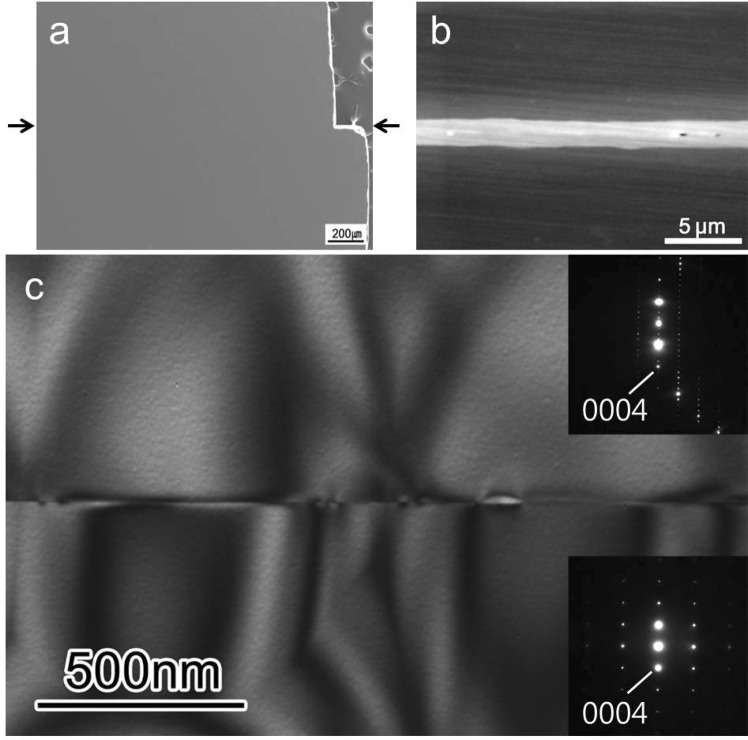
(**a,b**) SEM images of SiC bicrystal, and (**c**) bright-field TEM image of the grain boundary. The arrow indicates the grain boundary of the SiC bicrystal. Selected area electron diffraction patterns associated with the upper and lower crystals are shown in the insets. The lower crystal is viewed along the [$1\bar{1}00$] zone axis; the upper crystal is viewed along the direction approximately 3° degrees off the [$1\bar{2}10$] axis around the [0001] axis.

**Figure 2:**
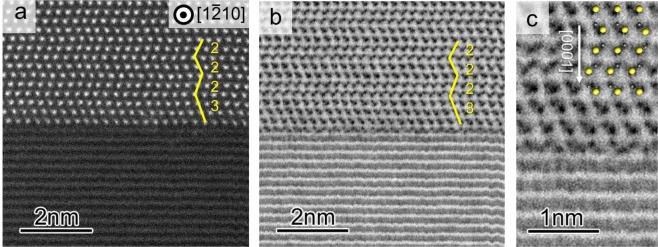
Microscopic analysis of the as-prepared SiC bicrystal: (**a**) HAADF-STEM image and (**b**) ABF-STEM image obtained simultaneously, and (**c**) magnified ABF-STEM image. The atomic structure of 4H-SiC is overlaid, where yellow and gray spheres correspond to Si and C atoms, respectively. The upper crystal is viewed along the [$1\bar{2}10$] zone axis, and atomic columns are clearly observed. In the lower crystal, only (0001) lattice fringes are observed because the projection axis is approximately 3° degrees off the [$1\bar{1}00$] axis. In the HAADF-STEM image, Si columns appear as bright spots. In the ABF-STEM image, Si and C columns appear as dark spots. Thus, the direction of [0001] axis can be strictly determined to be downward in the upper crystal (where the Si face is defined as the (0001) face). The yellow lines with numbers indicate the stacking sequence along the [0001] axis.

No visible defects at the grain boundary (Fig. 1a,b) were observed in the scanning electron microscopy (SEM) images of the SiC bicrystal joined by diffusion bonding. However, Fig. 1b (which is a higher magnification image of Fig. 1a) shows a thick interface between the SiC crystals. A typical bright-field TEM image of the SiC bicrystal (Fig. 1c) shows that the two crystals were well joined over a wide area. The diffraction patterns indicated that the lower crystal faced the [$1\bar{1}00$] axis, whereas the upper crystal faced a direction approximately 3° off the [$1\bar{2}10$] axis around the [0001] axis. The grain boundary was parallel to the (0001) plane of each crystal. Thus, the orientation of the present grain boundary could be expressed as a (0001)/[0001] 27° twist type. Note that the sign of the [0001] axis was determined by annular bright field-scanning transmission microscopy (ABF-STEM). Figure 2a,b show the simultaneously obtained high-angle annular dark field (HAADF)-STEM and ABF-STEM images, where the upper crystal was observed along the [$1\bar{2}10$] zone axis. In the HAADF image, Si columns of the upper crystal appeared as bright spots, and the horizontal fringes in the lower crystal corresponded to (0001) SiC molecular layers. It was evident that the grain boundary was atomically flat and parallel to the (0001) plane. The stacking sequence of 4H-SiC along the [0001] axis was (2, 2) according to Zhdanov notation, whereas the stacking of “3” was formed adjacent to the grain boundary, as indicated in Fig. 2a. In the ABF image, the Si and C columns appeared as dark contrasts, as observed in Fig. 2c. The positive direction of the [0001] axis was found to be downward in the upper crystal and was the same in the lower crystal (Fig. S1 and Supplementary Information). It is noted that the (0001) layer below the stacking of “3” showed periodic contrasts. These contrasts indicated the formation of an ordered atomic structure at the grain boundary without any amorphous phases.

**Figure 3:**
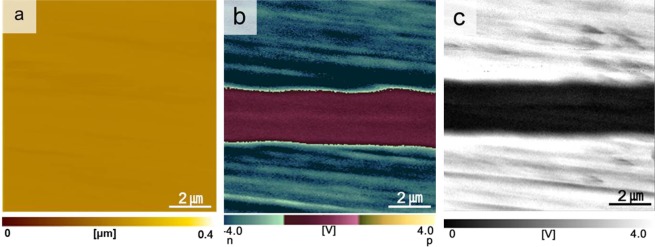
SPM images of the SiC bicrystal evaluated by SNDM: (**a**) topographic image, (**b**) carrier type image, and (**c**) carrier concentration image.

The topographic carrier type and carrier concentration images across the grain boundary were evaluated by scanning probe microscopy (SPM)-SNDM to clarify how the grain boundary affected the electrical conductivity of SiC. As shown in Fig. 3a, the topographic image seemed uniform, indicating a flat surface. However, the carrier type and carrier concentration images (Fig. 3b,c) were not homogeneous, indicating a clear interface effect. Both single crystals in the bicrystal showed n-type electrical conductivity. However, no n-type conductivity response was found near the grain boundary and in the darker area of the SEM image. A carrier depletion layer was present around the grain boundary with a width of approximately 1.8–2.4 μm.

A clear contrast was noticeable between the grain boundary area and the single crystals in the SEM image (Fig. 1b). This was consistent with the carrier depletion layer observed in the SPM-SNDM images (Fig. 3b,c). The width of the carrier depletion layer was 1.8–2.4 μm depending on the location with respect to the grain boundary. The formation of the carrier depletion layer could be explained by the following scenarios:

1. During solid-state diffusion bonding, the native oxide film (SiO_2_) of the SiC single crystals acted as a bonding phase between the two single crystals.
2. After the bonding of the two single crystals, oxygen atoms diffused to the SiC single crystals in both directions, resulting in the formation of an electrically resistive interfacial layer near the grain boundary.

**Figure 4:**
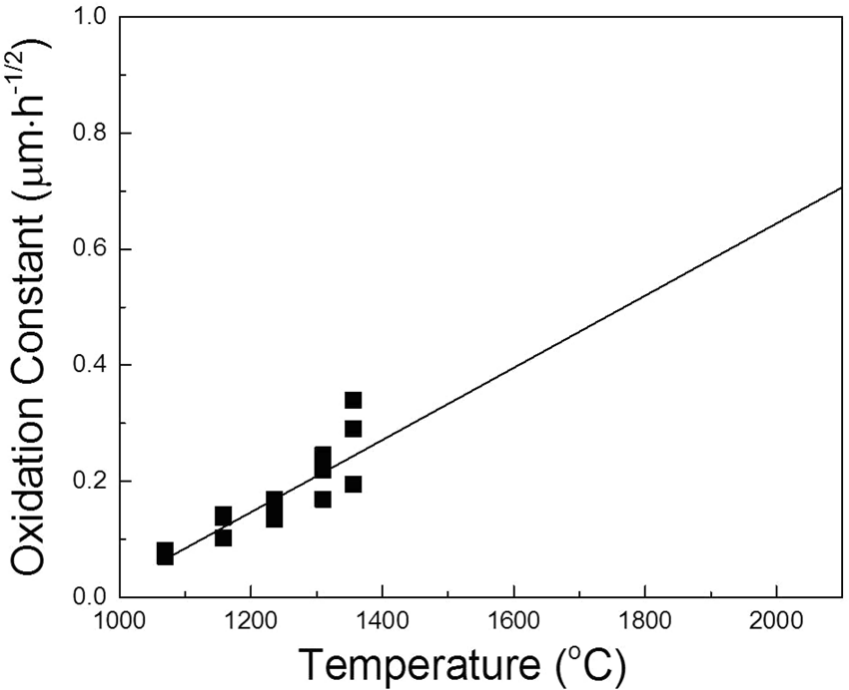
Oxygen diffusivity in the SiC lattice as a function of temperature. The literature data were in the range of 1.36 × 10^−18^–1.83 × 10^−18^ m^2^/s at 1070 °C and 1.06 × 10^−17^–3.21 × 10^−17^ m^2^/s at 1356 °C^23^. The oxygen diffusivity in the SiC lattice at 2050 °C was estimated from the extrapolation of existing data.

The oxygen diffusivity in the SiC lattice was 1.36 × 10^−18^–1.83 × 10^−18^ m^2^/s at 1070 °C and 1.06 × 10^−17^–3.21 × 10^−17^ m^2^/s at 1356 °C^23^. Because oxygen diffusivity was not observed in the SiC lattice at the solid-state diffusion bonding temperature (2050 °C), its value was estimated by extrapolation of the existing data (Fig. 4). Using the estimated value (1.39 × 10^−16^ m^2^/s at 2050 °C), the diffusion distance from the grain boundary after 2 h of diffusion bonding was calculated to be ~1 μm. Because the diffusion proceeded from the grain boundary in two directions in a two-dimensional image, the thickness of the interface layer was ~2 μm, which was nearly identical to the width of the observed carrier depletion layer (Fig. 3b,c).

**Figure 5:**
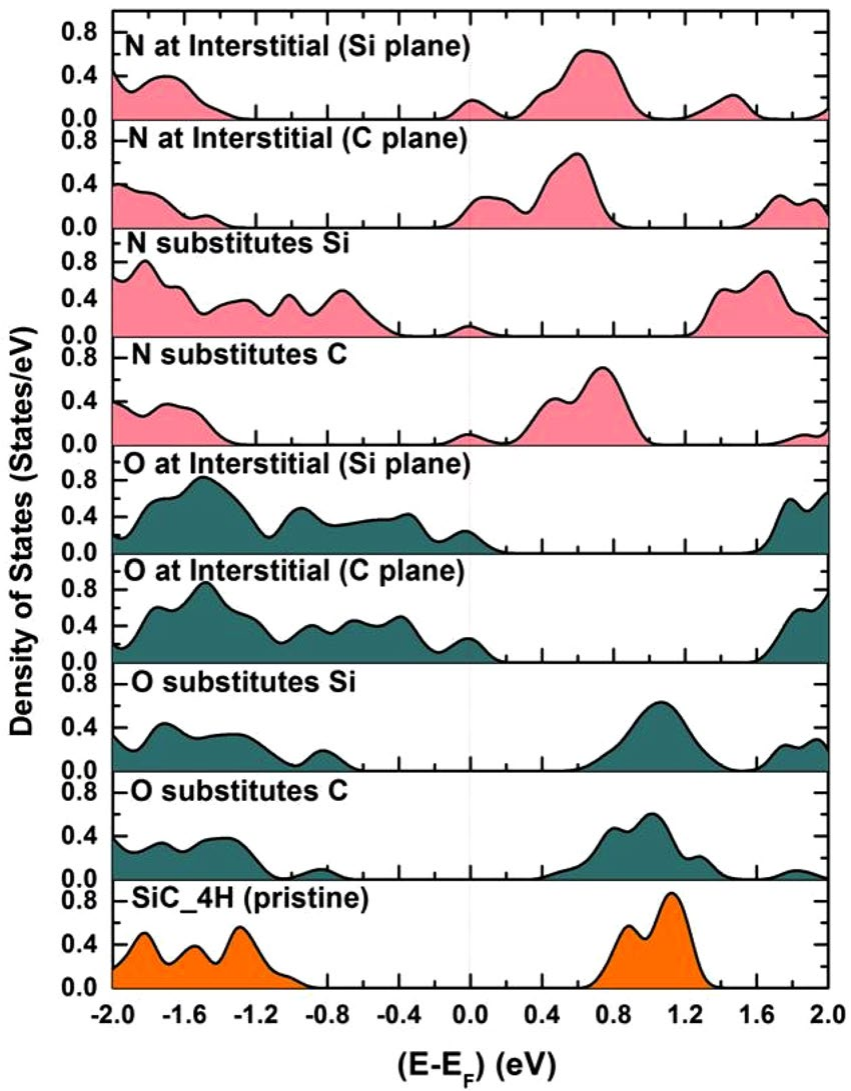
DOS of 4H-SiC when doped with either oxygen or nitrogen.

Figure 5 shows the density of states (DOS) of 4H-SiC calculated by DFT for different combinations of O and N doping. Although the effect of O and N atoms on the electronic structure of SiC depended on the specific bond formation, considering their electronegativities, the O and N atoms were expected to dope the holes and electrons of the sample, respectively. This behavior was observed for O at interstitial positions in Si or C planes that create holes, whereas for N doping, we observed a tendency of electron doping in both interstitials and C substitution. This analysis revealed that it was plausible to expect that oxygen acceptors will capture free carrier electrons provided by the nitrogen donors, thus contributing to the formation of an insulating depletion layer at the interface.

To confirm the aforementioned scenario and calculations, the oxygen contents in the bright area (interface) and SiC single crystal were analyzed using energy dispersive X-ray spectroscopy. As expected, the oxygen content in the bright area (0.82 ± 0.06 at%) was slightly higher than that in the SiC single crystal (0.71 ± 0.07 at%). The SEM (Fig. 1b) and TEM (Fig. 2) results were not consistent. A clean boundary was observed in the TEM images (Fig. 2), whereas a clear contrast was observed in the SEM image (Fig. 1b). The oxygen concentration differences between the bright region and SiC single crystal was only 0.11 at%. However, the incorporation of an additional 0.11 at% oxygen in the interface area transformed the conductive n-type SiC single crystal to a more electrically resistive material at the interface, as evidenced by the SPM-SNDM results (Fig. 3). It seemed that the higher concentration of oxygen impurities in the interfacial area created a potential barrier to electronic transport, thus affecting both SEM^24^ and carrier concentration images. Thus, the observed clear contrast in the SEM images of the interface area and single crystal (Fig. 1b) was caused by the different electrical conductivities in the interface area and SiC crystal. It should be noted that a polished bicrystal without any conductive layer coating was used as the specimen for SEM.

**Figure 6:**
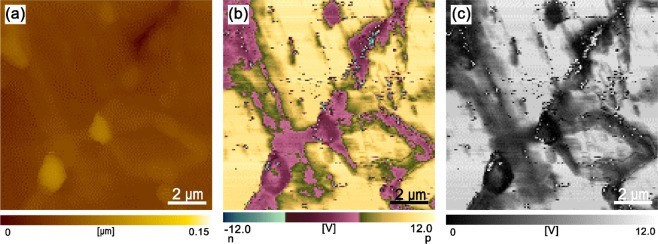
SPM images of the polycrystalline SiC evaluated by SNDM: (**a**) topographic image, (**b**) carrier type image, and (**c**) carrier concentration image.

To justify the current results in polycrystalline SiC ceramics, a polycrystalline SiC ceramic was fabricated by hot-pressing a SiC powder mixture containing 1 vol% Y_2_O_3_-Sc_2_O_3_ as additives. STEM-EDS analysis on the intergranular films of the polycrystalline SiC confirmed that nitrogen and oxygen atoms as well as other cationic atoms from additives and a native oxide film of SiC particles (Y, Sc, and Si) were segregated at the SiC-SiC boundaries^25^. The topographic carrier type and carrier concentration images across the grain boundary were evaluated by SPM-SNDM to clarify how the grain boundary affected the electrical conductivity of the polycrystalline SiC ceramic. As shown in Fig. 6a, the topographic image is uniform, indicating a flat surface. All grains in the polycrystalline SiC showed p-type electrical conductivity (Fig. 6b). However, the carrier type and carrier concentration images (Fig. 6b,c) show the existence of a carrier depletion layer near the grain boundary. Carrier depletion layers were present around the grain boundaries with a width of approximately 0.5–1.2 μm. The p-type carriers were attributed to the acceptors derived from Sc substitution in the Si sites of the SiC lattice^6,26^. The Sc in the additive composition incorporated into SiC lattice during sintering and acted as p-type dopants^27^. At present, there is insufficient evidence to establish the cause of the carrier depletion layer formation near grain boundaries of p-type polycrystalline SiC ceramics. Since nitrogen segregation was detected on the grain boundaries of the polycrystalline SiC^25^, nitrogen diffusion from the grain boundaries into SiC grains would be a possible scenario for the formation of carrier depletion layer in p-type polycrystalline SiC. Diffusion of nitrogen in SiC lattice is extremely slow^28^, however, significantly enhanced diffusion of nitrogen was reported in p-type doped SiC^29^.

Carrier depletion layers have also been observed at the grain boundaries of polycrystalline AlN-SiC solid solutions^19^ and have been estimated to exist at the epitaxial cerium oxide thin film/Al_2_O_3_ substrate interface^20^. Fehrer *et al*. [100] and other researchers [101,102] also suggested that electrical properties of GaN layers are mainly determined by potential barriers which are localized at grain boundaries. Based on previous results and this study, the grain boundary itself is believed to have little effect on the electrical conductivity of polycrystalline ceramics with clean grain boundaries (i.e., an absence of amorphous intergranular films). However, the carrier depletion layer formed near the grain boundary plays a critical role in electrical conductivity. Nevertheless, the width of the carrier depletion layer was found to be very different from that of the grain boundary.

## Conclusions

Defect-free SiC bicrystals with an n-type carrier were fabricated by solid-state diffusion bonding at 2050 °C and a pressure of 40 MPa in an argon atmosphere. STEM analysis demonstrated that the grain boundary showed a (0001)/[0001] 27° twist-type orientation and was atomically flat without any secondary phases such as an amorphous phase.

We have shown visually the existence of carrier depletion layer near the grain boundaries in both n-type SiC bicrystal and p-type polycrystalline SiC, using SPM-SNDM and STEM techniques. The carrier depletion layer was formed by the diffusion of oxygen from the native oxide film to the SiC single crystal and probably by the diffusion of nitrogen from the grain boundaries to the SiC grains in a SiC bicrystal and a polycrystalline SiC, respectively. These results suggest that the electrical conductivity of the SiC ceramics was mostly affected by the presence of a carrier depletion layer near the grain boundary and that the influence of the grain boundary itself was negligible when the grain boundary was clean (i.e., in the absence of an amorphous phase).

## Methods and Calculations

Commercially available n-type monocrystalline 4H-SiC wafers with a surface plane of (0001) and two-inch single-crystal ingots (Tankeblue Semiconductor Co. Ltd., Beijing, China) were used for this experiment. The micropipe density and surface roughness of the wafer were < 1 cm^−2^ and <0.2 nm, respectively. To characterize carrier distribution near the grain boundary using SNDM, SiC bicrystals were prepared through solid-state diffusion bonding of the two SiC wafers (10 × 10 × 1 mm3) at 2050 °C for 2 h under a pressure of 40 MPa in an argon atmosphere. To prepare polycrystalline SiC with 1 vol% of equimolar Y_2_O_3_-Sc_2_O_3_ additives, 98.63 wt% β-SiC (grade BF-17, H. C. Starck, Berlin, Germany), 0.85 wt% Y_2_O_3_ (99.99% pure, Kojundo Chemical Lab Co. Ltd., Sakado-shi, Japan), and 0.52 wt% Sc_2_O_3_ (99.99% pure, Kojundo Chemical Lab Co. Ltd.) were mixed by ball milling using SiC media in a polypropylene jar for 24 h in ethanol. The mixture was dried, sieved, and hot pressed at 2050 °C for 6 h under 40 MPa of pressure in a nitrogen atmosphere. The dopant distribution in both bicrystal and polycrystalline SiC was evaluated using a SPM-SNDM unit (SPA400, SII Nano Technology Inc., Chiba, Japan) in air with a Rh-coated cantilever (SI-DF40-R, SII Nano Technology). A bias voltage of 15 V_AC_ was applied between the cantilever and sample, and the change in capacitance was observed. Specimens for the SEM observations were prepared by cutting, grinding, and polishing the 10 × 10 × 2 mm^3^ bicrystal and polycrystalline SiC, and observations were performed without any conductive layer coating on the samples. Specimens for STEM observations were prepared by cutting, grinding, and argon ion-beam thinning of the 10 × 10 × 2 mm^3^ bicrystal. The grain boundaries of the TEM specimens of bicrystal were characterized by conventional TEM (JEM-2100HC, 200 kV, JEOL) and STEM (ARM-200F, 200 kV, JEOL). STEM images were acquired using a probe-forming semi-angle of 24 mrad and two annular detectors spanning a range 12–24 mrad (ABF) and 90–370 mrad (HAADF). The oxygen content in the bright area and SiC single crystal was analyzed using SEM with EDS (SU8010, Hitachi Ltd., Hitachi, Japan) with a probe size of 0.21 μm.

DFT calculations were performed to study the bandgap changes in SiC due to N or O doping. A systematic doping by the substitution of N/O at Si/C sites and at the interstitial positions in the Si/C plane was considered. We used a first-principles *ab initio* code Quantum Espresso^30,31^ as the basis of plane waves^32^ within the local density approximation^33^. We considered a super cell of 2 × 2 × 3 with 96 atoms of bulk 4H-SiC. The doping level was maintained at nearly 1% for each case. The structure was relaxed for each doping case until the forces on each atom were reduced to nearly zero (~10^−5^ Ry/Å). The DOS for each case was obtained to compare the effect of dopants on SiC. The obtained DOS were normalized by being divided with the number of atoms in a given super cell.

## Supplementary information

Supplementary Information
