# Supplementary material for: Carrier Depletion near the Grain Boundary of a SiC Bicrystal
Source: Sci Rep. 2019 Nov 29;9:18014. doi: 10.1038/s41598-019-54525-z (PMC6884474; doi:10.1038/s41598-019-54525-z)
Supplement: Supplementary file 1 — Supplementary Information [file 41598_2019_54525_MOESM1_ESM.docx]

**Carrier Depletion near the Grain Boundary of a SiC Bicrystal**

Young-Wook Kim^1^, Eita Tochigi^2^, Junichi Tatami^3^, Yong-Hyeon Kim^1^, Seung Hoon Jang^1^, Srivani Javvaji^4^, Jeil Jung^4^, Kwang Joo Kim^5^ & Yuichi Ikuhara^2^

^1^ Functional Ceramics Laboratory, Department of Materials Science and Engineering, The University of Seoul, Seoul 02504, Republic of Korea. ^2^ Institute of Engineering Innovation, The University of Tokyo, Tokyo 113-8656, Japan. ^3^Graduate School of Environmental and Information Sciences, Yokohama National University, Yokohama 240-9501, Japan. ^4^ Department of Physics, The University of Seoul, Seoul 02504, Republic of Korea. ^5^ Department of Physics, Konkuk University, Seoul 05029, Republic of Korea. Correspondence and requests for materials should be addressed to Y.W.K. (email: ywkim@uos.ac.kr)

**Supplementary Information**

*Additional STEM analysis of the grain boundary*

Figures S1a and S1b show simultaneously obtained HAADF- and ABF-STEM images, where the lower crystal is observed along the [1$\bar{1}$00] zone axis. In the HAADF image, Si columns in the lower crystal are imaged as bright spots. In the ABF image, atomic columns are imaged as dark spots. The Si and C columns are not resolved with each other because their projected separation distance (0.063 nm) is shorter than the spatial resolution of the ARM-200F (~0.08 nm). The relative position of the adjacent Si and C columns can be identified by image intensity. Figure S1c shows a line profile of image intensity associated with a (0001) layer, which was obtained by averaging the horizontal signals indicated by the box in the figure. The minimum point corresponding to the position of the Si column is located below the middle line, and the upper side has a gentler slope (i.e., is darker). This suggests that the C column is located above the Si column. Consequently, the positive direction of the [0001] axis is downward in both the lower and upper crystals.


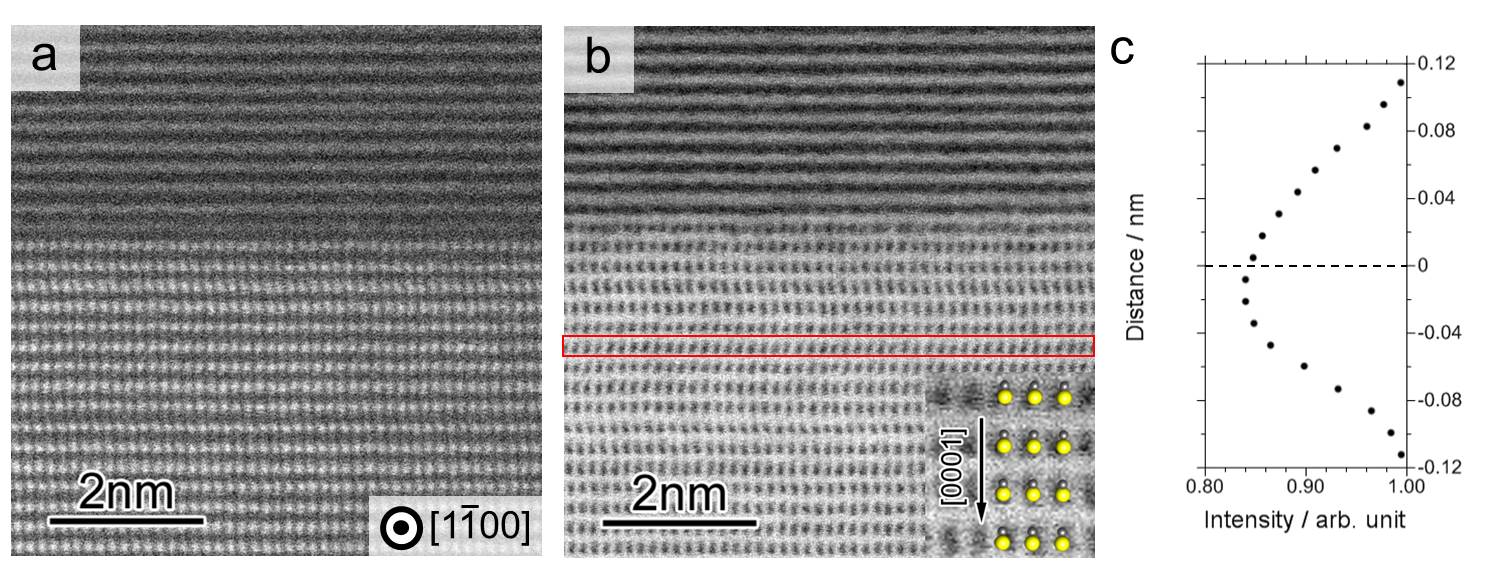


Fig. S1. (a) HAADF STEM image and (b) ABF STEM image obtained simultaneously. A magnified image with the atomic structure model of 4H-SiC is shown in the inset in (b). The lower crystal is viewed along the [1$\bar{1}$00] axis. (c) Line profile of image intensity obtained by averaging the horizontal signals within the red box in (b). The width of the red box is |1/4[0001]| ~ 0.25 nm.
